# Pre-operative MRI features predict early post-operative recurrence of hepatocellular carcinoma with different degrees of pathological differentiation

**DOI:** 10.1007/s11547-023-01601-0

**Published:** 2023-02-10

**Authors:** Zhi-ying Mo, Pei-yin Chen, Jie Lin, Jin-yuan Liao

**Affiliations:** 1 Department of Radiology, The First Affiliated Hospital of Guangxi Medical University, No. 6 Shuangyong Road, Nanning, 530021 Guangxi People’s Republic of China; 2 Department of Bone Surgery, Wuzhou Peopleʹs Hospital, No. 139 Sanlong Road, Wuzhou, 543000 Guangxi China

**Keywords:** Hepatocellular carcinoma, Pathological grading, Gadoxetic acid-enhanced MRI, Recurrence, Predictor

## Abstract

**Purpose:**

To investigate the value of pre-operative gadoxetate disodium (Gd-EOB-DTPA) enhanced MRI predicting early post-operative recurrence (< 2 years) of hepatocellular carcinoma (HCC) with different degrees of pathological differentiation.

**Methods:**

Retrospective analysis of pre-operative MR imaging features of 177 patients diagnosed as suffering from HCC and that underwent radical resection. Multivariate logistic regression assessment was adopted to assess predictors for HCC recurrence with different degrees of pathological differentiation. The area under the curve (AUC) of receiver operating characteristics (ROC) was utilized to assess the diagnostic efficacy of the predictors.

**Results:**

Among the 177 patients, 155 (87.5%) were males, 22 (12.5%) were females; the mean age was 49.97 ± 10.71 years. Among the predictors of early post-operative recurrence of highly-differentiated HCC were an unsmooth tumor margin and an incomplete/without tumor capsule (*p* = 0.037 and 0.033, respectively) whereas those of early post-operative recurrence of moderately-differentiated HCC were incomplete/without tumor capsule, peritumoral enhancement along with peritumoral hypointensity (*p* = 0.006, 0.046 and 0.004, respectively). The predictors of early post-operative recurrence of poorly-differentiated HCC were peritumoral enhancement, peritumoral hypointensity, and tumor thrombosis (*p* = 0.033, 0.006 and 0.021, respectively). The AUCs of the multi-predictor diagnosis of early post-operative recurrence of highly-, moderately-, and poorly-differentiated HCC were 0.841, 0.873, and 0.875, respectively. The AUCs of the multi-predictor diagnosis were each higher than for those predicted separately.

**Conclusions:**

The imaging parameters for predicting early post-operative recurrence of HCC with different degrees of pathological differentiation were different and combining these predictors can improve the diagnostic efficacy of early post-operative HCC recurrence.

## Introduction

Hepatocellular carcinoma (HCC) is the sixth most frequent cancer globally, fourth most lethal malignancy, and the second most prevalent cause of cancer-related mortality in China [1, 2]. Radical tumor resection is the preferred treatment for this disease [3]; however, the postoperative recurrence rate can reach 50–70% within 5 years [4]. Early recurrence (ER), defined as intrahepatic tumor occurrence within 2 years after surgical resection [5, 6], is predominantly attributable to the dissemination of the primary HCC and correlated with tumor-related factors such as microvascular invasion (MVI), worse histological differentiation, and microsatellite nodules [7–10]. Nevertheless, these risk factors are usually determined after surgical resection, restricting their utility in directing more active surgery (such as wider resection) or other treatments. Hence, preoperatively recognizing prognostic markers of HCC ER has long been of interest.

Imaging technologies have widely been applied to predict the HCC prognosis [11, 12]. Compared with dynamic enhanced CT and conventional MRI, gadolinium ethoxybenzyl diethylenetriamine pentaacetic acid (Gd-EOB-DTPA) enhanced MRI can provide more details of radiological HCC features, resulting in a higher lesion detection rate, especially for tumors with a diameter below 1 cm so that this technique provides more predictive information [13, 14]. Recently, studies on Gd-EOB-DTPA enhanced magnetic resonance imaging (MRI) have reported that several imaging features, such as peritumoral parenchymal enhancement, peritumoral hypointensity in the hepatobiliary phase (HBP), absence of a tumor capsule, tumor size, and non-smooth tumor margin have been proposed as significant predictors of early HCC recurrence [15–17]. In addition, with the widespread use of Liver Imaging Reporting and Data System (LI-RADS) v2018 in routine practice, recent studies have found that some standardized imaging features, including corona enhancement, mosaic architecture, and absence of fat in mass, were also independent recurrence predictors in high risk patients with HCC after hepatectomy [18–20].

The degree of tumor differentiation reflects the biological features of the tumor. The lesser the differentiation degree, the more invasive the tumor and the greater the probability of post-operative recurrence. The imaging features are able to reflect, at least to a certain extent, the growth pattern, invasiveness, and other biological characteristics of the tumor. Therefore, pre-operative imaging parameters of early post-operative recurred HCC with different degrees of pathological differentiation should be different. However, up to now, there is no study reporting on early recurrence stratified prediction based on pre-operative imaging features showing HCC of different degrees of pathological differentiation.

Therefore, we propose to assess the relationship of pre-operative Gd-EOB-DTPA-enhanced MRI findings with early post-operative HCC relapse and to predict the value of early recurrence of post-operative HCC with various degrees of pathological differentiation. As well, assessing the diagnostic efficacy of each predictor might result in a risk stratification assessment system for HCC patients and references for personalized precision treatments.

## Materials and methods

### Patients

**Fig. 1:**
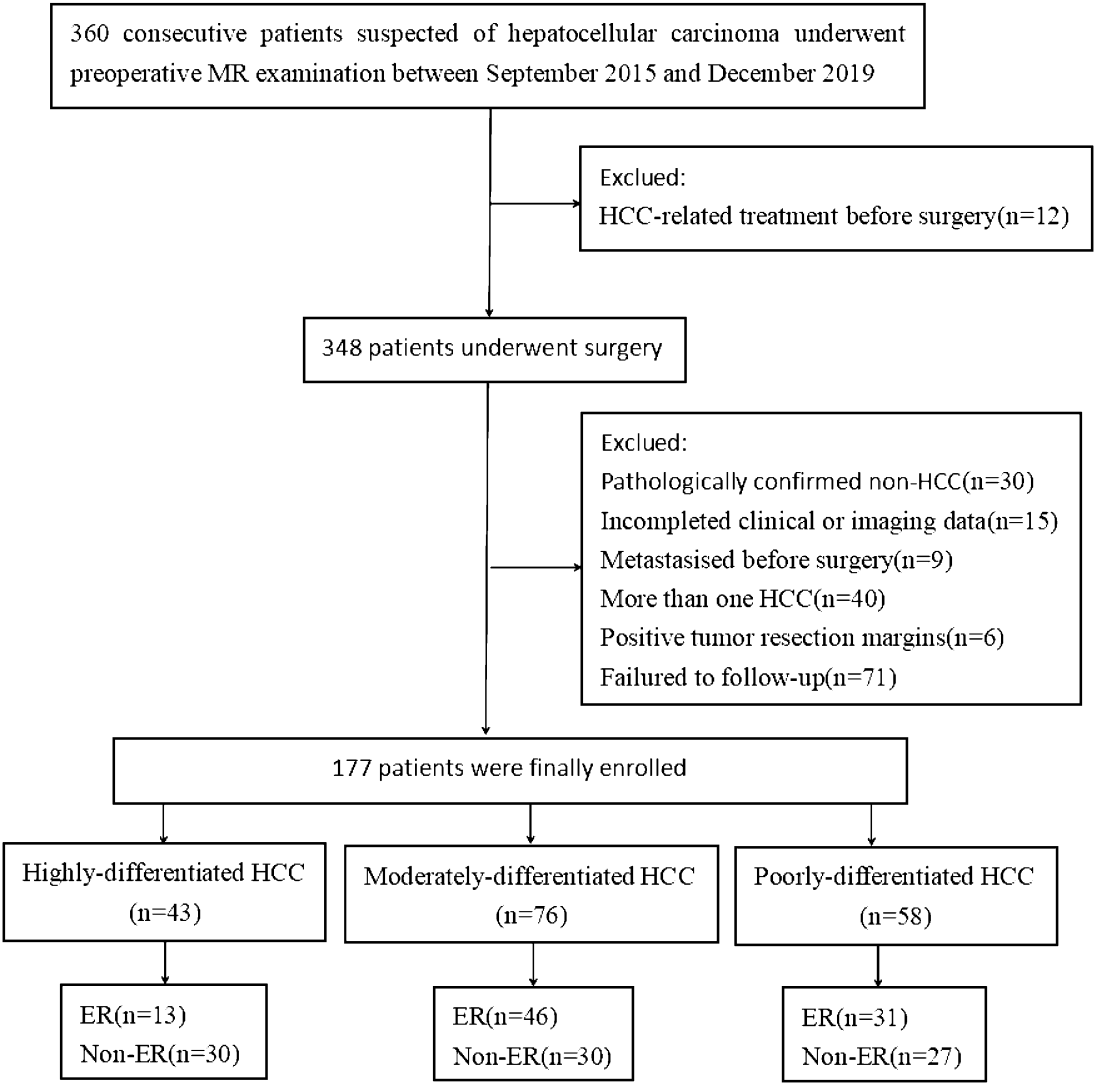
The flowchart shows the study group inclusion process. Numbers in parentheses are numbers of patients. HCC: Hepatocellular carcinoma, MRI: magnetic resonance imaging

This is a retrospective study in accordance with the Declaration of Helsinki and was approved by the Institutional Review Board (Approve Number: 2022-E431-01) of our hospital that waived the requirement of an informed consent. Between January 2015 and December 2019, a total of 360 consecutive high-risk patients were suspected of having HCC by ultrasound (US) or CT and underwent further preoperative gadoxetic acid-enhanced MRI at our institution. The following were the inclusion criteria: (1) All patients underwent radical resection of HCC (R0 resection) without any prior antitumoral therapies; (2) Gd-EOB-DTPA-enhanced MRI examination was performed within 2 weeks prior to resection; (3) Postoperative pathologic examination showed a single HCC, and (4) Regular follow-up within 2 years after surgery was performed. Figure 1 summarizes the flowchart of the research work. According to the postoperative pathological results these patients were categorized into three groups: a highly-differentiated HCC group, a moderately-differentiated HCC group and a poorly-differentiated HCC group. These groups were further divided into an early recurrence group and a non-recurrence group according to whether recurrence occurred within 2 years after surgical resection.

### MR techniques

All patients underwent hepatectomy within 2 weeks after the Gd-EOB-DTPA (Bayer Healthcare Co., Ltd., Germany)-enhanced MRI using a Magnetom Verio 3.0 T MR scanner (German Siemens) linked to an 8-channel body-phased array coil. Subjects were instructed to fast for 6 h prior to MR. The scanning settings along with the sequence were as follows: coronal T2WI (TR 1800.00 ms, TE 95.00 ms) along with half Fourier single excitation fast spin echo axial T2WI (TR 2930.00 ms, TE 89.00 ms) with the blade technology for axial fat suppression sequence T2WI (TR 2930.00 ms, TE 89.00 ms); 3-D reversal recovery fast gradient echo T1WI (TR 171.00 ms, TE 2.31 ms); single-pulse spin echo diffusion-weighted imaging (DWI) with scan b values of 0, 500, and 1000 s/mm^2^. All patients received a rapid bolus of 0.1 mL/kg (0.025 mmol/kg) of gadoxetic acid at a rate of 1.5 mL/s, immediately followed by a 20 mL saline flush through an antecubital venous catheter. The contrast medium was injected and scans of the arterial, portal venous, transitional, and HB phases performed at 20 s, 60 s, 180 s, and 20 min.

### Image analysis

All MRI images were independently evaluated by two radiologists (with 5 and 15 years of clinical experience in abdominal MR imaging, respectively), who were blinded with regard to the clinical and histopathological information. In case of a diagnostic disagreement, discussion and consultation were carried out aiming at reaching an agreement and highlighting all imaging characteristics useful for a prediction of early recurrence.

**Fig. 2:**
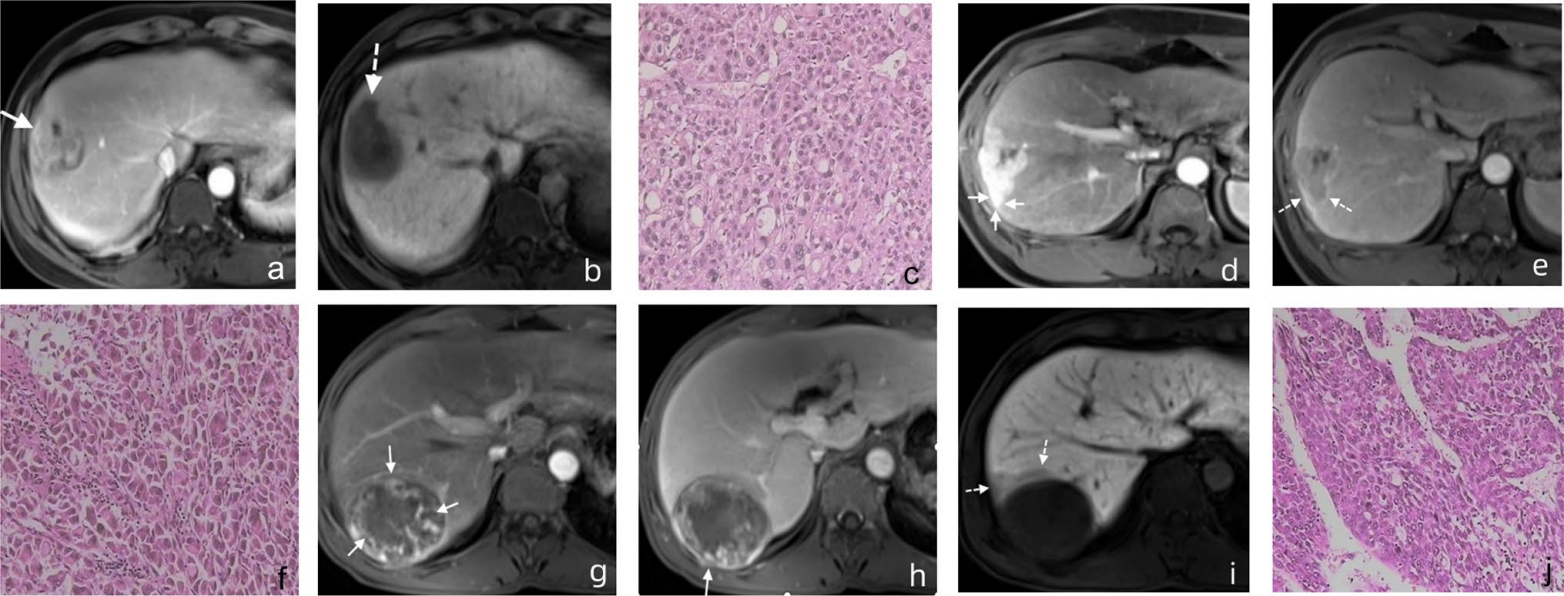
Typical cases of early post-operative HCC recurrence. **(a ~ c)** A 48-year-old male with highly differentiated HCC suffering from recurrent disease 18 months after surgery. Gd-EOB-DTPA-enhanced MRI showed an incomplete capsule enhancement in the portal venous phase (a, arrows) and the tumor margin in the hepatobiliary phase was not smooth but nodular (b, dashed arrow). The pathologic result of hematoxylin and eosin staining of a tumor tissue section (c) was highly-differentiated HCC. **(d ~ f)** A 38-year-old male with moderately differentiated HCC experienced recurrence 12 months after surgery. Gd-EOB-DTPA-enhanced MRI showed patchy enhancement areas around the tumor in the arterial phase (d, arrows) and an incomplete capsule enhancement at the tumor margins in the portal venous phase (e, dashed arrows). The pathologic result of hematoxylin and eosin staining of a tumor tissue section (f) was medium-differentiated HCC. **(g ~ j)** A 48-year-old female with poorly differentiated HCC and recurrence 9 months after surgery. In the arterial phase of the MRI, the mass showed inhomogeneously hyperenhancement (g, arrows); The portal venous phase showed washout, non-enhanced necrotic area and an incomplete capsule (h, arrow); The hepatobiliary phase showed an irregular peritumoral hypointensity (i, dashed arrows). The pathologic result of hematoxylin and eosin staining of a tumor tissue section (j) was poorly-differentiated HCC

For each HCC lesion, the following imaging features, as defined in LI-RADS v2018 [21], were evaluated: (a) LI-RADS major features: tumor size (≥ 20 vs. < 20 mm), nonrim arterial phase hyperenhancement, nonperipheral washout and enhancing capsule; and (b) LI-RADS ancillary features (favoring HCC in particular): nonenhancing capsule, nodule-in-nodule architecture, mosaic architecture, fat in mass, more than that of adjacent liver tissue, and blood products in mass. Additional non-LI-RADS imaging features that have been reported as potential predictors of HCC recurrence were also evaluated: (1) tumor margin was classified as smooth or unsmooth according to the presence of nodular protrusions extending into the surrounding liver parenchyma; (2) tumor capsule was defined as a thin, complete, or incomplete and enhanced rim around the tumor in the portal venous; (3) peritumoral parenchymal enhancement in the arterial phase, described as grossly hyperarterial contrast material enhancement beyond the tumor boundary that transforms into isointense with background liver parenchyma in the later dynamic phase images, irrespective of shape (for instance, wedge-shaped or circumferential); (4) peritumoral hypointensity in the HBP, described as an anomalous, wedge-shaped, or flame-like area exhibiting low signal intensity in the liver parenchyma outside the tumor margin in the hepatobiliary phase; (5) The maximum diameter of every tumor as determined with the help of electronic calipers on the image archiving and communication system (with a maximum diameter < 5 cm or ≥ 5 cm[4]); (6) intralesional necrosis was classified as present or absent; (7) tumor thrombosis, defined as a filling defect with enhancement in the portal or hepatic vein observed at the portal venous phase. Some representative cases are shown in Fig. 2.

### Clinical data

The analyzed clinical data comprised gender, age, α-fetoprotein (AFP) level (≤ 400 or > 400 ng/mL[16]), hepatitis B surface antigen (HBsAg) or hepatitis C surface antigen (HCsAg) (positive or negative) as well as the Child–Pugh grade.

### Histopathology evaluation

All original hepatic specimens were reviewed by two hepatic pathologists with more than 10 years of experience in hepatic pathology and who were blinded with regard to the imaging findings.

Pathological results analyzed in this study were histological differentiation, MVI, and cirrhosis. The Edmondson-Steiner’s system was applied to categorize all lesions into highly, moderately, and poorly differentiated groups [22]. When mixed grades co-existed within a tumor, the most severe grade was used. MVI was defined as the presence of cancer cell nests within a vascular space lined by endothelial cells that were visible only by microscopy [23].

### Follow-up surveillance after surgery

All patients were regularly followed up after discharge. Serum AFP levels, liver function tests, and ultrasound of the abdomen were conducted to monitor HCC recurrence during follow-up in the first month after liver resection and every 3 or 6 months thereafter. If there was an unexplained elevation of serum AFP-levels or new hepatic observations in US, CEUS, dynamic contrast-enhanced CT or MRI was performed for further evaluation. Early recurrence was defined as intrahepatic recurrence within the first 2 years after curative resection of HCC. Intrahepatic recurrence was defined as newly found intrahepatic tumors (with typical HCC imaging features [24, 25] or confirmed by pathology). If recurrence occurred after the first 2 years or no recurrence was detected during follow-up, the endpoint of the study was determined as “no early recurrence”.

### Statistical analysis

Descriptive quantitative variables were expressed as mean values ± standard deviations and categorical variables as frequencies and percentages. To compare variables between the patients in the early recurrence and the non-early recurrence group, we used the Student’s *t*-test for continuous variables and the χ^2^ or Fisher exact test for categorical variables. The forward LR approach was utilized to enter variables with a *P* < 0.05 in a univariate assessment into a stepwise logistic regression for a multivariate assessment. We adopted multivariate logistic regression to find independent indicators of early post-operative HCC relapse in subjects with varying degrees of pathological differentiation. ROC curve assessment was utilized to assess the diagnostic suitability of the predictors for early post-operative HCC recurrence with different degrees of differentiation. Differences in diagnostic performances were analyzed by comparing the ROC curves using log-rank tests. Statistical evaluation was conducted using SPSS 25.0 (SPSS Inc., Chicago, IL, U.S.A.) and MedCalc Statistical Software v15.8 (MedCalc Software bvba, Ostend, Belgium; https://www.medcalc.org). A two-tailed *P* < 0.05 was regarded as being statistically significantly different from controls. All confidence intervals (CI) are stated at the 95% level.

## Results

### Patient characteristics

The characteristics of the patients with different degrees of pathological differentiation in the ER and non-ER groups are summarized in Table 1. A total of 177 patients with solitary HCC were included in the study. Of the 177 patients (155 males and 22 females, mean age 49.97 ± 10.71; range 24 to 77 years), 90 had a recurring disease whereas the remaining 87 did not show recurrence within 2 years after surgery. The number of patients that had a recurring disease, displayed a highly, moderately, and poorly differentiated HCC with an incidence of 13, 46, and 31, respectively. The number of patients without recurrence was 30, 30, and 27, respectively.

**Table 1:** Patient characteristics in the ER and non-ER groups of HCC patients with different degrees of pathological differentiation

Characteristics	Overall HCC			Highly-differentiated HCC			Moderately-differentiated HCC			Poorly-differentiated HCC		
	ER(n = 90,%)	Non- ER(n = 87,%)	*P*	ER(n = 13,%)	Non- ER(n = 30,%)	*P*	ER(n = 46,%)	Non- ER(n = 30,%)	*P*	ER(n = 31,%)	Non- ER(n = 27,%)	*P*
*Gender*												
Male	79(87.8)	76(87.4)	0.932	12(92.3)	27(90.0)	1.000	39(84.8)	27(90.0)	0.756	28(90.3)	22(81.5)	0.554
Female	11(12.2)	11(12.6)		1(7.7)	3(10.0)		7(15.2)	3(10.0)		3(9.7)	5(18.5)	
Age (years)	50.19 ± 10.99	49.59 ± 10.32	0.707	56.69 ± 10.36	54.23 ± 8.99	0.436	49.93 ± 10.30	49.20 ± 10.32	0.762	47.84 ± 11.48	44.85 ± 9.73	0.294
*Cirrhosis*												
Yes	51(56.7)	47(54.0)	0.724	4(30.8)	19(63.3)	0.102	29(63.0)	13(43.3)	0.091	18(58.1)	15(55.6)	0.847
No	39(43.3)	40(46.0)		9(69.2)	11(36.7)		17(37.0)	17(56.7)		13(41.9)	12(44.4)	
*HBsAg/HCsAg*												
Positive	85(94.4)	78(89.7)	0.238	13(100)	25(83.3)	0.301	44(95.7)	26(86.7)	0.325	28(90.3)	27(100)	0.240
Negative	5(5.6)	9(10.3)		0(0)	5(16.7)		2(4.3)	4(13.3)		3(9.7)	0(0)	
*AFP*												
≤ 400	67(74.4)	72(82.8)	0.178	13(100)	30(100)	1.000	36(78.3)	28(93.3)	0.150	18(58.1)	14(51.9)	0.635
> 400	23(25.6)	15(17.2)		0(0)	0(0)		10(21.7)	2(7)		13(41.9)	13(41.9)	
*Child–Pugh*												
A	86(95.6)	84(96.6)	1.000	12(92.3)	29(96.7)	1.000	44(95.7)	28(93.3)	1.000	30(96.8)	27(100)	1.000
B	4(4.4)	3(3.4)		1(7.7)	1(3.3)		2(4.3)	2(6.7)		1(3.2)	0(0)	
*MVI*												
Yes	43(47.8)	21(24.1)	0.001*	0(0)	3(10.0)	0.542	21(45.7)	6(20.0)	0.022*	22(71.0)	12(44.4)	0.041*
No	47(52.2)	66(75.9)		13(100)	27(90.0)		25(54.3)	24(80.0)		9(29.0)	15(55.6)	

*ER* early recurrence, *Non-ER* non early recurrence, *HCC* hepatocellular carcinoma, *AFP* alpha-fetoprotein, *MVI* microvessel invasion

^*^p < 0.05

Sixty-four patients displayed MVI (n = 64/177, 36.2%) with a prevalence for the highly, moderately, and poorly differentiated HCC of 7.0%, 35.5%, and 58.6%, respectively. In patients with early recurrence, significantly more MVI was observed (47.8%, 43/90) compared to patients with non-early recurrence (24.1%, 21/87, *P* = 0.001). The MVI rate attained statistical significance in the moderately and poorly differentiated HCC subgroup between the recurrence and the non-recurrence cohort. The MVI rate was not significantly different in the highly differentiated HCC subgroup between the recurrence and the non-recurrence cohort.

There were no significant differences in age, gender, cirrhosis, HBV/HCV infection, AFP, and Child–Pugh classification between the relapse group and the non-relapse group in subjects with overall, highly, moderately, or poorly differentiated HCC (*P* > 0.05).

### Univariate analysis

Univariate analysis demonstrated that the following parameters resulted in statistically significant differences between the recurrence and the non-recurrence cohort: for the overall HCC, these parameters were tumor size, tumor margin, tumor capsule, peritumoral enhancement, peritumoral hypointensity, intralesional necrosis, and tumor thrombosis. For the highly-differentiated HCC, these parameters were tumor margin and tumor capsule. For the moderately-differentiated HCC subgroup, these parameters were tumor size, tumor capsule, peritumoral enhancement, peritumoral hypointensity, intralesional necrosis, and tumor thrombosis and finally, for the poorly-differentiated HCC, these parameters were peritumoral enhancement, peritumoral hypointensity, and tumor thrombosis. However, there were no significant differences in all LI-RADS features between the two groups in the overall, highly, moderately, or poorly differentiated HCC group (Table 2).

**Table 2:** Relationship between MRI features and early post-operative HCC recurrence with different degrees of pathological differentiation

Characteristics	Overall HCC			Highly-differentiated HCC			Moderately- differentiated HCC			Poorly-differentiated HCC		
	ER(n = 90,%)	Non-ER(n = 87,%)	*P*	ER(n = 13,%)	Non-ER(n = 30,%)	*P*	ER(n = 46,%)	Non-ER(n = 30,%)	*P*	ER(n = 31,%)	Non-ER(n = 27,%)	*P*
Non-LIRADS imaging features												
Tumor size(cm)												
≥ 5	43(47.8)	24(27.6)	0.006*	3(23.1)	5(16.7)	0.945	26(56.5)	10(33.3)	0.048*	14(45.2)	9(33.3)	0.358
< 5	47(52. 2)	63(72.4)		10(76.9)	25(83.3)		20(43.5)	20(66.7)		17(54.8)	18(66.7)	
Peritumoral enhancement												
Yes	57(63.3)	15(17.2)	0.000*	4(30.8)	4(13.3)	0.356	32(69.6)	6(20.0)	0.000*	21(67.7)	5(18.5)	0.000*
No	33(36.7)	72(82.8)		9(69.2)	26(86.7)		14(30.4)	24(80.0)		10(32.3)	22(81.5)	
Tumor capsule												
Complete	13(14.4)	42(48.3)	0.000*	1(7.7)	18(60.0)	0.002*	5(10.9)	17(56.7)	0.000*	7(22.6)	7(25.9)	0.766
Incomplete/Without	77(85.6)	45(51.7)		12(92.3)	12(40.0)		41(89.1)	13(43.3)		24(77.4)	20(74.1)	
Tumor margin												
Smooth	20(22.2)	39(44.8)	0.001*	2(15.4)	20(66.7)	0.002*	11(23.9)	13(43.3)	0.075	7(22.6)	6(22.2)	0.974
Unsmooth	70(77.8)	48(55.2)		11(84.6)	10(33.3)		35(76.1)	17(56.7)		24(77.4)	21(77.8)	
Peritumoral hypointensity												
Yes	52(57.8)	10(11.5)	0.000*	1(7.7)	3(10.0)	1.000	31(67.4)	3(10.0)	0.000*	20(64.5)	4(14.8)	0.000*
NO	38(42.2)	77(88.5)		12(92.3)	27(90.0)		15(32.6)	27(90.0)		11(35.5)	23(85.2)	
Intralesional necrosis												
Present	65(72.2)	45(51.7)	0.005*	6(46.2)	13(43.3)	0.864	36(78.3)	16(53.3)	0.022*	23(74.2)	16(59.3)	0.227
Absent	25(27.8)	42(48.3)		7(53.8)	17(56.7)		10(21.7)	14(46.7)		8(25.8)	11(40.7)	
Tumor thrombosis												
Present	24(26.7)	5(5.7)	0.000*	1(7.7)	1(3.3)	1.000	9(19.6)	0(0)	0.010*	14(45.2)	3(11.1)	0.011*
Absent	66(73.3)	82(94.3)		12(92.3)	29(96.7)		37(80.4)	30(100)		17(54.8)	24(88.9)	
LI-RADS major features												
Tumor size(cm)												
< 2 cm	10(11.1)	18(20.7)	0.081	3(23.1)	9(30.0)	0.925	6(13.0)	3(10.0)	0.970	1(3.2)	6(22.2)	
≥ 2 cm	80(88.9)	69(79.3)		10(76.9)	21(70.0)		40(87.0)	27(90.0)		30(96.8)	21(77.8)	0.070
Nonrim APHE												
Present	89(98.9)	80(92.0)	0.063	13(100)	27(90.0)	0.542	46(100)	28(93.3)	1.531	30(96.8)	25(92.6)	0.902
Absent	1(1.1)	7(8.0)		0(0)	3(7.0)		0(0)	2(6.7)		1(3.2)	2(7.4)	
Washout												
Present	89(98.9)	83(95.4)	0.344	13(100)	28(93.3)	1.000	45(97.8)	29(96.7)	1.000	31(100)	26(96.3)	0.466
Absent	1(1.1)	4(4.6)		0(0)	2(6.7)		1(2.2)	1(3.3)		0(0)	1(3.7)	
Enhancing capsule												
Present	81(90.0)	82(94.3)	0.295	12(92.3)	28(93.3)	1.000	40(87.0)	28(93.3)	0.615	29(93.5)	26(96.3)	1.000
Absent	9(10.0)	5(5.7)		1(7.7)	2(6.7)		6(13.0)	2(6.7)		2(6.5)	1(3.7)	
LI-RADS ancillary features(favoring HCC in particular)												
Nonenhancing capsule^a^ Mosaic architecture												
Present	7(7.8)	9(10.3)	0.552	1(7.7)	3(10.0)	1.000	4(8.7)	2(6.7)	1.000	2(6.5)	4(14.8)	0.541
Absent	83(92.2)	78(89.7)		12(92.3)	27(90.0)		42(91.3)	28(93.3)		29(93.5)	23(85.2)	
Nodule-in-nodule architecture												
Present	3(3.3)	7(8.0)	0.302	0(0)	2(6.7)	1.000	2(4.3)	2(6.7)	1.000	1(3.2)	3(11.1)	0.508
Absent	87(96.7)	80(92.0)		13(100)	28(93.3)		44(95.7)	28(93.3)		30(96.8)	24(88.9)	
Fat in mass												
Present	4(4.4)	5(5.7)	0.958	1(7.7)	3(10.0)	1.000	2(4.3)	2(6.7)	1.000	1(3.2)	0(0)	1.000
Absent	86(95.6)	82(94.3)		12(92.3)	27(90.0)		44(95.7)	28(93.3)		30(96.8)	27(100)	
Blood products												
Present	18(20.5)	9(10.3)	0.064	1(7.7))	1(3.3)	1.000	10(21.7)	4(13.3)	0.534	7(22.6)	4(14.8)	0.677
Absent	70(79.5)	78(89.7)		12(92.3)	29(96.7)		36(78.3)	26(86.7)		24(77.4)	23(85.2)	

*ER* early recurrence, *Non-ER* non early recurrence, *HCC* hepatocellular carcinoma, *LI-RADS* Liver Imaging Reporting and Data System, *APHE* arterial phase hyperenhancement

^*^p < 0.05

^a^None of the observations had the feature of nonenhancing capsule

### Multivariate logistic regression analysis

The statistically remarkable predictors of the univariate assessment were incorporated in the multivariate logistic regression model. The data showed that incomplete/without tumor capsule (OR = 15.707; 95% CI:2.362,12.693; *p* = 0.001), peritumoral enhancement (OR = 8.596; 95% CI:1.540,8.787; *p* = 0.003), peritumoral hypointensity (OR = 9.476; 95% CI:1.697,10.836; *p* = 0.002), and tumor thrombosis (OR = 5.805; 95% CI:1.318,14.705; p = 0.016) were predictors for early post-operative recurrence in this 177 HCC patients group. The data also showed that an unsmooth tumor margin (OR = 6.817; 95% CI:1.121,41.467; *p* = 0.037) along with an incomplete/without tumor capsule (OR = 11.483; 95% CI:1.211,108.879; *p* = 0.033) were predictors for early post-operative recurrence of a highly-differentiated HCC. We also found that an incomplete/without tumor capsule (OR = 7.416; 95% CI:1.881,48.274; *p* = 0.006), peritumoral enhancement (OR = 3.969; 95% CI:1.024,16.239; *p* = 0.046), along with peritumoral hypointensity (OR = 8.416; 95% CI:2.171,54.831; *p* = 0.004), were predictors of early post-operative recurrence of moderately-differentiated HCC. For the poorly-differentiated HCC patient subgroup, we identified peritumoral enhancement (OR = 4.554; 95% CI:1.139,21.362; *p* = 0.033), peritumoral hypointensity (OR = 7.625; 95% CI:1.913,45.743; *p* = 0.006), and tumor thrombosis (OR = 5.343; 95% CI:1.349,38.098; *p* = 0.021) as predictors for an early post-operative recurrence (Table 3).

**Table 3:** Multivariate logistic regression of the predictors for early post-operative HCC recurrence with different pathological differentiation stages

Predictor	*β*	OR	95% CI	*P*
Highly-differentiated HCC				
Incomplete/Without tumor capsule	2.441	11.483	1.211 ~ 108.879	0.033*
Unsmooth tumor margin	1.919	6.817	1.121 ~ 41.467	0.037*
Moderately-differentiated HCC				
Incomplete/Without tumor capsule	2.254	7.416	1.881 ~ 48.274	0.006*
Peritumoral enhancement	1.491	3.969	1.024 ~ 16.239	0.046*
Peritumoral hypointensity	2.390	8.416	2.171 ~ 54.831	0.004*
Poorly-differentiated HCC				
Peritumoral enhancement	1.596	4.554	1.139 ~ 21.362	0.033*
Peritumoral hypointensity	2.236	7.625	1.913 ~ 45.743	0.006*
Tumor thrombosis	1.970	5.343	1.349 ~ 38.098	0.021*

*HCC* hepatocellular carcinoma, *β* regression coefficient, *CI* confidence interval, *OR* odds ratio

*p < 0.05

### Evaluation of the multi-predictor diagnosis efficacy of early post-operative HCC recurrence with different degrees of pathological differentiation

**Fig. 3:**
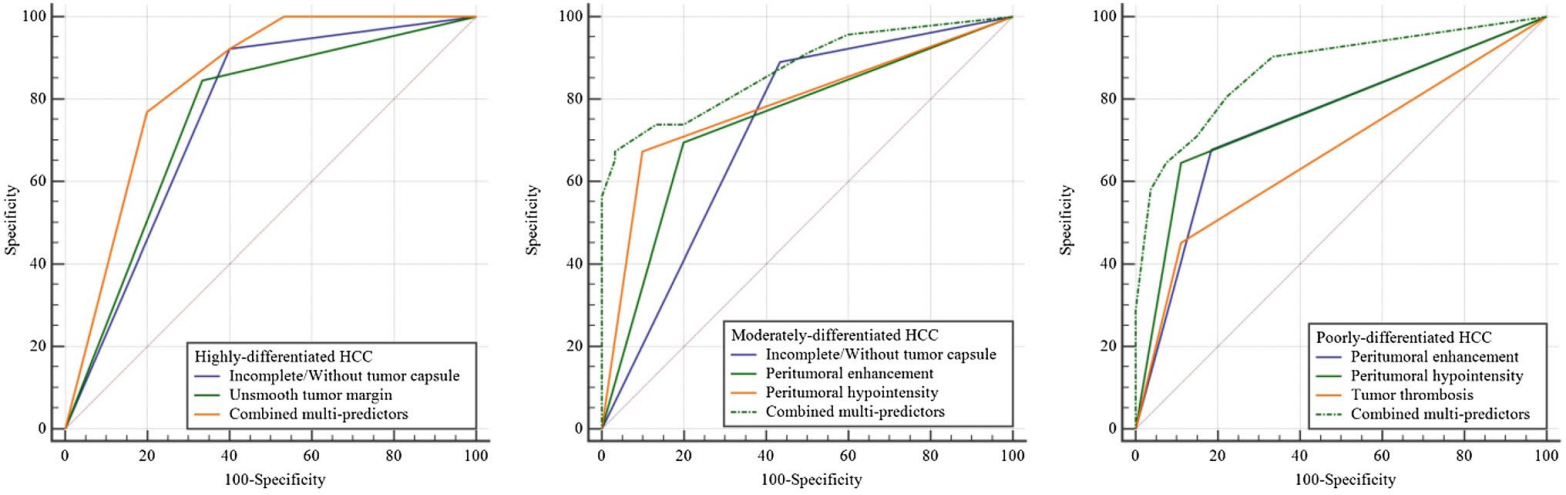
The ROC curves of the independent predictors for early post-operative HCC recurrence with different degrees of pathological differentiation

By combining multiple HCC predictors, we could show a high diagnostic predictivity (Table 4, Fig. 3). For the prediction of an early post-operative recurrence with a highly-differentiated phenotype, the AUCs of the unsmooth tumor margin and incomplete/without tumor capsule were 0.756 and 0.762, the diagnostic performance improved up to 0.841 (95% CI: 0.697,0.934) with a sensitivity of 76.9% along with a specificity of 80.0% by the combined use of these two predictors data. For the prediction of an early post-operative recurrence with a moderately-differentiated HCC, the AUCs of incomplete/without tumor capsule, the peritumoral enhancement, and the peritumoral hypointensity were 0.729, 0.748, and 0.787. By combining these three predictors data, the diagnostic performance improved up to 0.873 (95% CI: 0.777,0.938) with a sensitivity of 67.4%, as well as a specificity of 93.4%. For the prediction of an early post-operative recurrence with a poorly-differentiated HCC, the AUCs of the peritumoral enhancement, the peritumoral hypointensity, and the tumor thrombosis were 0.746, 0.767, and 0.670. When we combined these three predictors data, the diagnostic performance improved up to 0.875 (95% CI: 0.787,0.962) with a sensitivity of 80.7% along with a specificity of 77.8%. The AUC of the combined predictors was significantly higher than the AUC of the single predictors. The difference was statistically significant (*P* < 0.05).

**Table 4:** Diagnostic efficacy of the predictors for early post-operative HCC recurrence with different degrees of pathological differentiation

Predictor	AUC	Sensitivity(%)	Specificity(%)	95%CI	*P*
Highly-differentiated HCC					
Incomplete/Without tumor capsule	0.762	92.3	60.0	0.607 ~ 0.878	< 0.001
Unsmooth tumor margin	0.756	84.6	66.7	0.602 ~ 0.874	< 0.001
Combined multi-predictors	0.841	76.9	80.0	0.697 ~ 0.934	< 0.001
Moderately-differentiated HCC					
Incomplete/Without tumor capsule	0.729	89.1	56.7	0.615 ~ 0.825	< 0.001
Peritumoral enhancement	0.748	69.6	80.00	0.635 ~ 0.840	< 0.001
Peritumoral hypointensity	0.787	67.4	90.00	0.678 ~ 0.873	< 0.001
Combined multi-predictors	0.873	67.4	93.4	0.777 ~ 0.938	< 0.001
Poorly-differentiated HCC					
Peritumoral enhancement	0.746	67.7	81.2	0.615 ~ 0.851	< 0.001
Peritumoral hypointensity	0.767	64.5	88.9	0.662 ~ 0.872	< 0.001
Tumor thrombosis	0.670	45.2	88.9	0.563 ~ 0.778	0.002*
Combined multi-predictors	0.875	80.65	77.8	0.787 ~ 0.962	< 0.001

*HCC* hepatocellular carcinoma, *AUC* area under the curve, *CI* confidence interval

*p < 0.05

## Discussion

In this study, Gd-EOB-DTPA-enhanced MRI signals could not only predict the early post-operative recurrence of HCC but also stratify the risk of different degrees of pathological differentiation. Our study demonstrated that an incomplete/without tumor capsule, peritumoral enhancement, peritumoral hypointensity, and tumor thrombosis were the predictors of solitary HCC ER. Among the predictors of highly-differentiated HCC ER were unsmooth tumor margin and incomplete/without tumor capsule whereas those of moderately-differentiated HCC ER were incomplete/without tumor capsule and peritumoral enhancement along with peritumoral hypointensity. The predictors of poorly-differentiated HCC ER were peritumoral enhancement, peritumoral hypointensity, and tumor thrombosis. Preoperative risk stratification is essential for clinicians to identify patients at increased risk of postoperative early recurrence, which may contribute to risk-based personalized disease management. This may include considering an extended resection or liver transplantation depending on the HCC image predictors with different pathological differentiation, or making tailored follow-up plans and subsequent adjuvant treatment strategies for them.

Previous studies have elucidated that an unsmooth tumor margin is an independent predictor of early post-operative HCC recurrence [16, 26]. In our study, an unsmooth tumor margin has been shown to be an independent predictor of early post-operative recurrence of a highly-differentiated HCC phenotype, but there was no significant difference between the recurrence group and the non-recurrence group for moderately- and poorly-differentiated HCC. The mechanism might be that the tumor margin characteristics are associated with HCC heterogeneity and aggressiveness. The tumor is prone to grow outwards with decreasing degrees of tumor differentiation, finally leading to the gradual emergence of unsmooth margins [27]. When highly-differentiated HCC shows irregular margins, this tumor tissue is rich in new blood vessels, which increases the likelihood of the cancer cells infiltrating the surrounding tissues and thus resulting in intrahepatic HCC metastasis and recurrence [28].

A complete tumor capsule is considered to be a favorable factor of reducing the post-operative HCC recurrence risk. [4, 29]. Previous studies have reported that HCCs with an incomplete tumor capsule showed a greater recurrence risk [27, 30], which is consistent with our study. Our further research showed that an incomplete tumor capsule is an independent predictor of the post-operative recurrence of highly- and moderately-differentiated HCC. However, in poorly-differentiated HCC, most of the tumor capsules were incomplete and no significant difference was found in the prediction suitability of the pseudocapsule between the recurrence and the non-recurrence group.

In previous studies [31, 32], it was reported that the peritumoral enhancement and hypointensity were predictors of post-operative HCC recurrence. We could not only confirm this but furthermore were able to specify this in a way that both parameters were predictors for early post-operative recurrence of moderately- and poorly-differentiated HCC both of which were associated with a high risk of microvascular invasion in a relatively poorly differentiated HCC. However, in highly-differentiated HCC, it is not easy to invade the surrounding liver parenchyma. In this research work, the incidence of peritumoral enhancement and hypointensity in both the early recurrence and the non-recurrence group of highly-differentiated HCC were low and there was no significant difference.

Vascular invasion, whether in great vessels or capillaries, is an expression pattern of the aggressive biological behavior of tumors. Tumor thrombus and MVI have been widely recognized as poor prognostic factors in patients undergoing radical hepatectomy [33–36]. Consistent with prior research, the incidence of MVI in the early recurrence group was significantly higher than that in the non-recurrence group in the present study. The lower degree of tumor differentiation was associated with a higher MVI incidence. In our study, multivariate regression analysis showed that a macroscopic vascular tumor thrombus on magnetic resonance images was an independent risk factor for postoperative recurrence in the poorly differentiated HCC situation. It was clear that the occurrence of a microcarcinoma thrombus or a macroscopic tumor thrombus could directly affect the patient's prognosis following hepatectomy, especially in those with low differentiated tumors.

Unlike prior studies [4, 37, 38], we did not find that tumor size and intralesional necrosis were independent predictors of ER, although they were significant influencing factors of ER in univariable analysis. Thus, whether tumor size and intralesional necrosis can predict early recurrence after HCC remains a key problem to be solved in future studies.

The LI-RADS major features and ancillary features (favoring HCC in particular) did not show significant associations with early recurrence in our study. However, some recent studies have reported that the absence of fat in mass, blood products in mass, and presence of mosaic architecture were independent predictors of recurrence [18, 20, 37]. This discrepancy may be attributed to the heterogeneity of the study population.

In our study, combining multiple HCC predictors led to an improvement in the diagnostic performance of the prediction models for the early post-operative recurrence of highly-, moderately-, and poorly-differentiated HCC with AUCs of 0.841, 0.873, and 0.875, respectively. The AUC of the combined predictors was significantly higher than the AUC of single predictors (the difference was statistically significant). We suggest that the combination of independent MR imaging features predicts HCC ER preoperatively more reliably compared to each independent MR imaging feature alone.

However, this study suffers from some limitations. First, the retrospective research work may have a selection bias. Second, we only enrolled patients with a single HCC; therefore, the results cannot be generalized to patients suffering from multiple tumors. Third, the sample size is unbalanced, with a small sample size of well-differentiated HCC, which may lead to incomplete MRI features, and thus, the sample size should be expanded. Fourth, the present study was a single-center study, however, a multi-center validation study would deliver more reliable results. Thus, future multicenter prospective studies with a larger patient population are warranted to confirm these promising results and hopefully may be able to establish a better preoperative prediction model with higher sensitivity.

## Conclusions

Gd-EOB-DTPA-enhanced MRI imaging features can be helpful in predicting an early post-operative HCC recurrence with different degrees of pathological differentiation. The combination of different independent predictors can improve the diagnostic efficacy of early post-operative HCC recurrence. In conclusion, the current study provides references to help clinicians better assess individual HCC patient survival prognoses.
